# Classifier-driven generative adversarial networks for enhanced antimicrobial peptide design

**DOI:** 10.1093/bib/bbaf500

**Published:** 2025-10-25

**Authors:** Michaela Areti Zervou, Effrosyni Doutsi, Yannis Pantazis, Panagiotis Tsakalides

**Affiliations:** Computer Science Department, University of Crete, University Campus, Voutes, 715 00, Heraklion, Greece; Institute of Computer Science, Foundation for Research and Technology-Hellas, Nikolaou Plastira 100, 700 13, Heraklion, Greece; Institute of Computer Science, Foundation for Research and Technology-Hellas, Nikolaou Plastira 100, 700 13, Heraklion, Greece; Institute of Applied and Computational Mathematics, Foundation for Research and Technology-Hellas, Nikolaou Plastira 100, 700 13, Heraklion, Greece; Computer Science Department, University of Crete, University Campus, Voutes, 715 00, Heraklion, Greece; Institute of Computer Science, Foundation for Research and Technology-Hellas, Nikolaou Plastira 100, 700 13, Heraklion, Greece

**Keywords:** protein de novo design, antimicrobial peptides, generative adversarial networks, large protein language models, transfer learning, multi-task learning

## Abstract

The development of antimicrobial peptides (AMPs) presents a promising approach to addressing antibiotic-resistant pathogens. Computational methods, such as Feedback Generative Adversarial Networks (FBGANs), have demonstrated strong performance in optimizing AMP design. FBGAN operates as a classifier-guided Generative Adversarial Network (GAN), refining training data by replacing them with the classifier’s most accurate predictions based on a predefined threshold. However, this method may introduce bias and constrain the diversity and quality of the generated peptides. To address these limitations, we propose a novel classifier-driven GAN (cdGAN) framework that seamlessly integrates classifier predictions into the generative model’s loss function. This enables an adaptive, end-to-end learning process that enhances AMP generation without requiring explicit data modifications. By embedding classifier guidance within the loss computation, cdGAN dynamically optimizes both peptide diversity and functionality. Comparative studies indicate that cdGAN outperforms conventional guided-GAN architectures, such as Conditional GANs and Auxiliary Classifier GANs, while achieving performance comparable to or exceeding established AMP design methods. Additionally, cdGAN’s flexible architecture allows for the simultaneous optimization of multiple peptide attributes. To demonstrate this capability, we introduce a multi-task classifier based on the Evolutionary Scale Modeling 2 (ESM2) model, enabling cdGAN to assess both antimicrobial activity and peptide structural properties in parallel. This enhancement improves the likelihood of generating viable therapeutic candidates with enhanced antimicrobial effectiveness and reduced toxicity.

## Introduction

Antimicrobial peptides (AMPs) are a promising alternative to traditional antibiotics due to their broad-spectrum activity and ability to target bacterial membranes, which reduces the likelihood of resistance development [1]. One of the key factors in AMP functionality is the presence of $\alpha $-helical structures, which enhance their amphiphilic nature that allows interaction with both hydrophobic and hydrophilic regions of bacterial membranes, leading to effective membrane disruption [2, 3]. However, therapeutic peptides must also exhibit minimal toxicity to human cells, particularly avoiding hemolysis, as excessive hemolytic activity can cause red blood cell destruction and severe side effects [4]. Many potent AMPs fail in clinical applications due to unintended cytotoxicity, limiting their therapeutic viability. These considerations highlight the importance of designing AMPs that balance antimicrobial potency, structural optimization, and safety [4].

To accelerate AMP discovery, deep learning approaches have been widely adopted, with Generative Adversarial Networks (GANs) emerging as a particularly promising tool [5–8]. GAN-based frameworks, such as Feedback GAN (FBGAN) [5], have demonstrated their ability to generate peptide sequences that closely mimic natural AMPs. The FBGAN model employs an additional classifier to guide the generator toward producing more AMP-like sequences based on a predefined threshold score. More recently, FBGAN-ESM2 [8] incorporated a pre-trained Evolutionary Scale Modeling 2 (ESM2) classifier [9] to enhance peptide selection, improving the antimicrobial efficacy of generated peptides.

Although effective, the general FBGAN framework relies on selecting sequences based on predefined AMP score thresholds, which can introduce bias and limit peptide diversity. By prioritizing high-scoring sequences, this model risks overfitting to known patterns, thereby reducing its ability to identify novel AMPs with unique properties. Furthermore, because classifier predictions are not always accurate, misclassified sequences may be included in training, resulting in incorrect or misleading data. These challenges emphasize the need for a more adaptive and unbiased generative approach.

**Figure 1:**
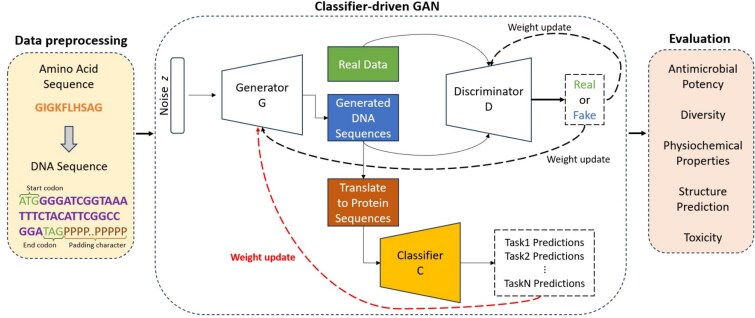
Architecture of the classifier-driven GAN (cdGAN) framework for AMP generation. The cdGAN framework integrates a generator (G), discriminator (D), and a multi-task classifier (C) to generate DNA-encoded antimicrobial peptide (AMP) sequences with biologically meaningful properties. In the preprocessing step, amino acid sequences are translated into DNA sequences using fixed-length encoding, including a start codon (ATG), a stop codon (TAG, TAA, or TGA), and a padding character P, which is constrained to appear only at the end of the sequence. The generator takes a noise vector $ z $ as input and produces synthetic DNA sequences. These sequences are evaluated by a discriminator, which attempts to distinguish between real and generated DNA sequences, providing feedback to improve the generator. Simultaneously, the DNA sequences are translated into protein sequences and passed through a classifier that predicts sequence-level properties relevant to antimicrobial peptide (AMP) functionality. The generator is trained using feedback from both the discriminator and the classifier, optimizing for both realistic sequence generation and the desired biological properties. Finally, evaluation metrics are applied to assess the quality of the generated sequences.

To address these issues, this work presents a classifier-driven GAN (cdGAN) framework that incorporates classifier predictions directly into the GAN loss function as provided in Fig. 1. Unlike traditional GANs, which rely on explicit selection of generated peptides, cdGAN allows for adaptive and continuous learning without data manipulation. This end-to-end approach reduces selection bias and improves model generalization, ultimately enhancing the diversity and novelty of generated AMPs. Moreover, by dynamically incorporating classifier feedback into the training process, cdGAN mitigates the risk of overfitting to predefined high-scoring peptides.

Beyond single-task antimicrobial design, cdGAN is extended to a multi-task framework that simultaneously optimizes antimicrobial activity and structural properties. Given the crucial role of $\alpha $-helical structures in AMP function, we develop a novel multi-task classifier based on ESM2 that evaluates both antimicrobial potential and structural characteristics. This multi-task learning strategy enhances peptide design by ensuring that generated AMPs possess not only potent antimicrobial properties but also optimal structural features for membrane interaction [2, 3].

Finally, to ensure the safety of generated peptides, we implement a rigorous post-generation screening step using ToxinPred 3.0 [10] and HemoPI [11] to filter out toxic and hemolytic candidates. This additional screening step significantly enhances the therapeutic viability of generated AMPs by prioritizing sequences that are both effective and safe for clinical applications.

## Materials and methods

### Datasets and preprocessing

This study compares our proposed model with various guided-GAN variants and state-of-the-art approaches for AMP design. To train both the proposed cdGAN and baseline guided-GAN models, we constructed a dataset of 5,200 protein sequences ranging from 10 to 50 residues, including 2600 AMPs from APD3 [12] and 2600 non-AMPs from reviewed UniProt entries [13]. Non-AMPs were clustered using MMseqs2 [14] with a 50 percent sequence identity threshold, and one representative per cluster was selected to ensure diversity. Pairwise Needleman-Wunsch alignments [15] showed an average sequence similarity of 0.2 between the AMP and non-AMP sets, limiting the usefulness of sequence alignment and encouraging the model to capture additional features. The diversity and physicochemical analyses (Tables 3, 5, and Fig. 6) support that the model learns biologically meaningful patterns beyond sequence similarity.

To enable multi-task classification, structural annotations were added using Porter 5 [16], which labels each sequence by secondary structure elements: helix (H), strand (E), and coil (C), while preserving the sequence length. Sequences with more than 10 consecutive helix (H) elements are classified as likely $\alpha $-helical structures [5]. This structural information is combined with AMP annotations, forming a multi-task classification dataset capturing both antimicrobial activity and potential structural features of the peptides.

To standardize sequence encoding and introduce biological diversity, each amino acid sequence was converted to a complementary DNA (cDNA) sequence by assigning codons based on known genetic coding. For amino acids with multiple codons, one codon was randomly selected to introduce natural variability, following the approach in [5]. The process of translating the amino acid sequence into DNA through random codon assignments is reversible and does not affect the final protein’s function [17, 18]. Finally, shorter sequences were padded with the character ‘P’ at the end to a uniform length of 156 nucleotides, ensuring input compatibility during training. An example is provided in Fig. 1.

### Guided generative adversarial networks

In this work, we refer to the FBGAN-ESM2 model, as well as Conditional GANs (cGANs) and Auxiliary Classifier GANs (ACGANs), as guided-GANs, since they incorporate task-specific labels to steer generation toward desired outcomes.

For the implementation, we adopt the Wasserstein GAN with Gradient Penalty (WGAN-GP) framework [19], known for its stability during training and its ability to produce high-quality samples. The core of the WGAN-GP approach involves minimizing the Wasserstein distance [20] between the real data distribution $ P_{r} $ and the distribution of generated samples $ P_{g} $ that is expressed as follows: 


\begin{align*} & W(P_{r}, P_{g}) = \sup_{\|D\|_{L} \leq 1} \mathbb{E}_{x \sim P_{r}}[D(x)] - \mathbb{E}_{x \sim P_{g}}[D(x)], \end{align*}

where $ \|D\|_{L} $ denotes the Lipschitz constraint on the discriminator $ D $. Here, $ \mathbb{E}_{x \sim P_{r}}[D(x)] $ and $ \mathbb{E}_{x \sim P_{g}}[D(x)] $ represent the expected discriminator outputs on real and generated data, respectively.

To ensure the stability of the training process and uphold the Lipschitz constraint, a gradient penalty term is introduced, improving the quality of generated samples and promoting smoother training dynamics [19].

#### Conditional generative adversarial networks

Conditional GANs (cGANs) [21] extend the GAN framework by conditioning the generation process on auxiliary information, such as class labels or desired attributes. In cGANs, both the generator and the discriminator receive this conditional input, which guides the generation of samples in a targeted manner.

The objective function for the discriminator in a Wasserstein cGAN with gradient penalty can be written as: 


\begin{align*} & L_{D} = \mathbb{E}_{x \sim P_{r}}[D(x|y)] - \mathbb{E}_{{x} \sim P_{g}}[D({x}|y)] \end{align*}

\begin{align*} &+ \lambda \mathbb{E}_{{x} \sim P_{\hat{x}}} \left[ (\|\nabla_{{x}} D({x}|y)\|_{2} - 1)^{2} \right], \end{align*}

where $ P_{\hat{x}} $ is the distribution of the interpolated samples between the real and generated data, and $ \lambda $ is the gradient penalty coefficient. The generator’s objective function is: 


\begin{align*} & L_{G} = -\mathbb{E}_{z \sim P_{z}}[D(G(z|y)|y)]. \end{align*}

Here, $ y $ represents the conditioning variable, and the generator aims to produce outputs consistent with this input.

#### Auxiliary classifier generative adversarial networks

Auxiliary Classifier GANs (ACGANs) [22] further enhance the conditional generation process by incorporating an auxiliary classifier within the discriminator architecture. This classifier is tasked with predicting the class labels associated with the generated samples, providing an additional layer of feedback during training.

The loss function for the discriminator in an Wasserstein ACGAN with gradient penalty comprises three components: the Wasserstein loss, the gradient penalty, and the classification loss: 


\begin{align*} & L_{D} = \mathbb{E}_{x \sim P_{r}}[D(x|y)] - \mathbb{E}_{{x} \sim P_{g}} [D(x|y)] \end{align*}

\begin{align*} &+ \lambda \mathbb{E}_{{x} \sim P_{\hat{x}}} \left[ (\|\nabla_{{x}} D({x}|y)\|_{2} - 1)^{2} \right] \end{align*}

\begin{align*} &+ \mathbb{E}_{x \sim P_{r}}[\log(Q(x|y))] + \mathbb{E}_{z \sim P_{z}}[\log(Q(G(z|y)|y))], \end{align*}

where $ Q $ is the auxiliary classifier predicting the label $ y $. The generator’s objective function is: 


\begin{align*} & L_{G} = -\mathbb{E}_{z \sim p_{z}(z)}[D(G(z|y)|y)] - \mathbb{E}_{z \sim P_{z}}[\log(Q(G(z|y)|y))]. \end{align*}

This dual-loss structure encourages the generator to create samples that closely resemble real data while also meeting specific label characteristics.

### Classifier-driven generative adversarial networks

This study introduces a novel classifier-driven Generative Adversarial Network (cdGAN) framework, designed to generate protein sequences with multiple desired properties. The cdGAN architecture extends the WGAN-GP model by incorporating a multi-task classifier that provides targeted feedback to guide the generative process.

The cdGAN consists of three core components: a generator $ G $, a discriminator $ D $, and a classifier $ C $. The generator synthesizes protein sequences from latent noise vectors, while the discriminator evaluates these sequences to distinguish between real and generated data. The classifier predicts the likelihood that the generated sequences exhibit specific target properties, and this feedback is integrated into the generator’s training, allowing it to produce sequences that meet defined biological criteria. To optimize multiple tasks simultaneously, the classifier output probabilities are multiplied, with a product closer to 1 indicating greater simultaneous satisfaction of all target properties. This product operator serves as a soft analogue to the logical AND operator, where all properties must be activated (i.e. set to ON) to have an effect. A logarithmic transformation is subsequently applied to enhance numerical stability and ensure the values are appropriately scaled within the overall loss function. The objective function of the cdGAN is expressed as: 


\begin{align*} \min_{G} \max_{D} \Big( &\mathbb{E}_{x \sim P_{r}}[D(x)] - \mathbb{E}_{z \sim P_{z}}[D(G(z))] \\ + & \ \lambda \, \mathbb{E}_{{x} \sim P_{\hat{x}}} \left[ \left( \| \nabla_{{x}} D({x}) \|_{2} - 1 \right)^{2} \right] \\ - & \sum_{t=1}^{N} a_{t} \cdot \mathbb{E}_{z \sim P_{z}}[\log C_{t}(G(z))] \Big) \end{align*}

where $ \lambda $ controls the strength of the gradient penalty, $ P_{\hat{x}} $ denotes the interpolated distribution between real and generated distributions, and $ C_{t}(G(z)) = P(C_{t}=1 | G(z)) $ represents the probability that the generated sample $ G(z) $ belongs to the target class for task $ t $, as assessed by the classifier $ C $. Each classification task $ t = 1, \dots , N $ is assigned a weight $ a_{t} $, adjusting the influence of each task’s feedback on the generator’s objective.

At the core of this framework is the multi-task classifier, which is developed using transfer learning to evaluate multiple characteristics of protein sequences. To achieve this, we chose the ESM2 model [9], which has demonstrated strong performance in antimicrobial peptide classification, with the ESM2-t12 variant selected specifically for its computational efficiency and effective balance of representational power [23]. The classifier is trained using a combined loss function to simultaneously optimize multiple tasks: 


\begin{align*} &{\mathcal{L}_{mlt}} = \sum_{t=1}^{N}\lambda_{t} \mathcal{L}_{t}, \end{align*}

where $t=1, \dots , N$ is the number of tasks, $ \mathcal{L}_{t}$ is the loss for each task, and $\lambda _{t}$ is a hyperparameter that determines the weight of each task’s loss, and it must satisfies $\lambda _{k} \ge 0$. In addition, the sum of all task-specific weights $ \lambda _{t} $ is constrained to $ \sum _{t} \lambda _{t} = 1 $, ensuring a balanced contribution of each task to the total loss. By adjusting the values of $ \lambda _{t} $, the model can prioritize certain tasks, allowing for flexible optimization and achieving balanced performance across all tasks.

## Results

In this section, we present the computational performance of each model. Technical details on model parameters, experimental settings, and hardware specifications are provided in the supplementary material.

### Classifier performance analysis

The classification of AMPs is important in drug design and the development of targeted therapies [5, 8, 24]. AMPs often adopt $\alpha $-helical structures, which are particularly advantageous due to their ability to disrupt cellular protective layers. Consequently, detecting the structural fold of an AMP provides valuable insights into its functional mechanisms.

Numerous classifiers have been designed to identify AMP properties [25–28], while others focus on classifying structural folds [16, 29–31]. A recent study proposed a multi-task learning framework leveraging Transformer networks and $k$-mers technique to classify both AMP properties and $\alpha $-helical structure [32] simultaneously. The $k$-mers method is applied on DNA sequences to provide meaningful representations, however, it requires careful optimization of $k$ to achieve a balance between sequence detail and computational efficiency.

**Figure 2:**
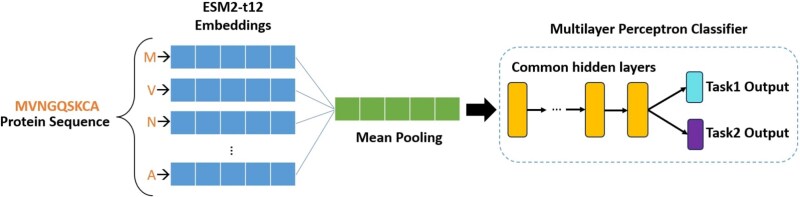
Overview of the proposed transfer learning-based architecture utilizing the ESM2 protein language model and a Multilayer Perceptron (MLP) classifier.

To eliminate the need for manual sequence preprocessing and to work directly at the protein level, we propose a novel multi-task classifier, illustrated in Fig. 2. Our model utilizes embeddings derived from the pre-trained ESM2 protein language model, which processes amino acid sequences directly, and feeds them into a lightweight Multilayer Perceptron (MLP) for efficient and scalable classification. The joint loss function for the two prediction tasks is defined as follows: 


\begin{align*}&{\mathcal{L}_{mlt}} = \lambda_{1} \mathcal{L}_{1} + \lambda_{2} \mathcal{L}_{2}\, \ \lambda_{2} = 1- \lambda_{1}, \ \lambda_{1}, \lambda_{2}\ge 0 \end{align*}

Table 1 compares the single-task classification performance of our proposed MLP-ESM2 classifier against the $k$-mers Transformer model for both AMP (Task 1) and $\alpha $-helical folding prediction (Task 2) independently. As demonstrated, the MLP-ESM2 classifier achieves significantly better performance compared to the $k$-mers-based model for both tasks as evaluated by three different metrics: the accuracy (ACC), the area under the curve (AUC), and the F1-score. The results indicate that the proposed MLP-ESM2 classifier provides a robust and computationally efficient approach to protein property prediction, achieving high accuracy, AUC, and F1 scores across both tasks. This suggests that leveraging transfer learning with ESM2 embeddings can significantly enhance classification performance in bioinformatics tasks while mitigating the complexity associated with training larger networks.

**Table 1:** Single-task classification results for AMP (Task 1) and $\alpha $-helical folding prediction (Task 2) using the proposed MLP-ESM2 classifier and a $k$-mers Transformer model. Standard deviations are reported in parentheses

	Task 1 ($\lambda _{1}=1$)		Task 2 ($\lambda _{2}=1$)	
	MLP-ESM2	$k$ -mers Transformer	MLP-ESM2	$k$ -mers Transformer
ACC	**91.6** (1.0)	81.1 (1.4)	**92.8** (0.8)	74.7 (1.2)
AUC	**97.1** (0.3)	88.2 (0.6)	**98.1** (0.3)	80.7 (1.3)
F1	**91.7** (1.0)	80.9 (1.7)	**92.8** (0.8)	80.2 (1.1)

The performance of the proposed multi-task MLP-ESM2 classifier across various $\lambda _{1}$ and $\lambda _{2}$ values is presented in the Supplementary Table 1. The optimal values were selected based on the Pareto front shown in Supplementary Figure 1. This figure illustrates that variations in $\lambda _{1}$ have minimal impact on overall classification performance, enabling robust classification despite shifts in task prioritization. Table 2 highlights the performance of the multi-task classifiers, demonstrating that our proposed model significantly outperforms the $k$-mers Transformer on both tasks.

**Table 2:** Performance comparison of the optimal $k$-mers Transformer ($\lambda _{1}=0.7$) and MLP-ESM2 ($\lambda _{1}=0.4$) multi-task classifiers in AMP (Task 1) and $\alpha $-helical folding prediction (Task 2)

	$k$ -mers Transformer		MLP-ESM2	
	Task 1	Task 2	Task 1	Task 2
ACC	79.9 (2.1)	75.9 (0.4)	**91.3 (0.7)**	**92.6 (0.8)**
AUC	88.3 (0.8)	82.0 (1.7)	**96.9 (0.5)**	**97.9 (0.2)**
F1	79.0 (3.5)	82.0 (0.5)	**91.4 (0.8)**	**93.3 (0.8)**

### Comparison of cdGAN with other guided-GANs

In this section, we analyse the proposed cdGAN model against baseline guided-GAN architectures, including cGANs, ACGANs, and FBGAN-ESM2. We generated 5000 sequences per model using the final trained generator, with random noise as input and, for conditional models, the appropriate AMP class label. These generated sequences were then used for all subsequent comparisons against real AMP sequences from reference databases. In particular, we evaluated their diversity and predicted antimicrobial potency. High diversity is crucial to ensure the generative model is not simply reproducing known sequences. For antimicrobial potency, we employed predictive models to estimate the biological activity of the generated sequences. This evaluation ensures that the generated peptides are not only diverse but also functionally relevant, aligning to discover new antimicrobial peptides.

The generated data were compared to real AMPs sourced from six widely recognized datasets: XUAMP [33], CAMP [34], dbAMP [35], DRAMP [36], LAMP [37], and YADAMP [38]. Duplicate sequences were removed, and only peptides with lengths between 10 to 50 amino acids were selected for analysis.

#### Diversity of generated peptides

**Figure 3:**
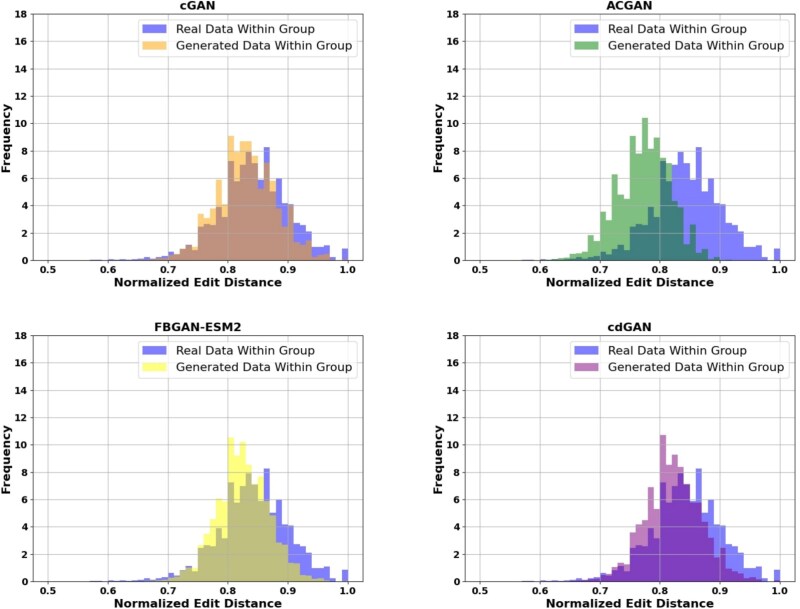
Normalized within group edit distance distribution for real and generated data, highlighting the fidelity and diversity of different models.

The edit distance analysis, shown in Fig. 3, provides insights into the diversity of the generated sequences. The normalized edit distance measures the difference between real and generated sequences by counting the minimum number of insertions, deletions, or substitutions required to transform one sequence into another, offering a way to assess how closely models capture the distribution of real peptides. As provided, the distribution of the generated data for cdGAN, FBGAN-ESM2, and cGAN aligns closely with the distribution of real data. In contrast, ACGAN shows the weakest alignment, with its generated distributions deviating significantly from the real data, highlighting challenges in capturing diversity.

Table 3 shows both training data similarity and in-between sequence similarity for each model. To highlight the classifier’s role in the GAN framework, we include a plain GAN, which follows the cdGAN architecture but is trained only on AMP sequences without classifier guidance. More details on the architectures can be found in the supplementary material.

**Table 3:** Percentage of sequence similarity and diversity within the generated data for each model, and novelty relative to the training data

	In-between Sequence Similarity	Diversity	Novelty
Real data	22.9%	100%	100%
cGAN	28.2%	100%	100%
ACGAN	40.3%	100%	100%
FBGAN-ESM2	27.6%	100%	100%
cdGAN (This work)	**27.4%**	100%	100%

For in-between sequence similarity, ACGAN shows the highest redundancy (40.3%), meaning its generated peptides are highly similar to each other. In contrast, cdGAN achieves the lowest in-between similarity (27.4%), followed closely by FBGAN-ESM2 (27.6%) and the plain GAN (27.6%), highlighting their ability to generate a more diverse set of peptides. Finally, all models exhibited 100% novelty, confirming that none of the generated sequences overlapped with the training set.

#### Antimicrobial property prediction

While the previous metrics provide valuable insights into the fidelity and diversity of generated sequences, they do not capture functional relevance. To address this, we evaluated the generated sequences using the CAMP$_{R4}$ platform [34], which predicts AMP activity through external classifiers such as Support Vector Machine (SVM), Random Forest (RF), and Artificial Neural Network (ANN). The classification accuracy for RF is 86.5%, 84.1% for SVM, and 82.2% for ANN. This step is essential for assessing how well the generated sequences generalize to unseen data and whether they retain key functional characteristics. By leveraging external classifiers trained on diverse datasets, we minimize biases introduced by our specific training data, ensuring a more objective and robust evaluation of the generated peptides’ applicability in real-world contexts.

The results summarized in Table 4, indicate that the RF classifier consistently identifies a higher proportion of sequences as AMPs across all models. Overall, ACGAN and cdGAN achieve the strongest performance; however, the high performance of ACGAN is likely attributed to the high sequence similarity of its generated peptides, which as shown in Table 3, is 40.3%. This increased similarity allows the generated sequences to align closely with patterns recognized by the classifiers, reflecting a trade-off between reduced diversity and enhanced classifier-predicted functionality. Moreover, the performance of ACGAN is not consistent across all classifiers, with the ANN-based classifier achieving approximately 10% lower accuracy since it relies on learned features, unlike RF and SVM, which benefit from direct sequence similarity. In contrast, cdGAN performs better with ANN classifiers, and given the flexibility of neural networks, there is significant potential for further improvements, which is not as easily achievable with RF and SVM. Overall, cdGAN demonstrates a more balanced performance across classifiers, maintaining high diversity while generating sequences with sufficient similarity to real AMPs. Lastly, GAN, cGAN, and FBGAN-ESM2 exhibit comparable performance, though they underperform compared to cdGAN and ACGAN.

**Table 4:** Percentage of predicted AMPs with CAMP$_{R4}$ platform equipped with Support Vector Machine (SVM), Random Forest (RF), and Artificial Neural Network (ANN) models for AMP prediction. The best performance per classifier is highlighted in bold, and the standard deviation is reported in parentheses

	CAMP$_{R4}$ predictions			
	RF	SVM	ANN	Average (std)
GAN	58.0%	51.5%	52.8%	54.1% (2.8)
cGAN	59.2%	56.1%	57.2%	57.5% (1.2)
ACGAN	**77.6%**	**72.2%**	65.6%	**71.8% (4.9)**
FBGAN-ESM2	59.1%	55.5%	58.9%	57.8% (1.6)
cdGAN (This work)	75.8%	68.6%	**70.5%**	71.6% (3.0)

**Figure 4:**
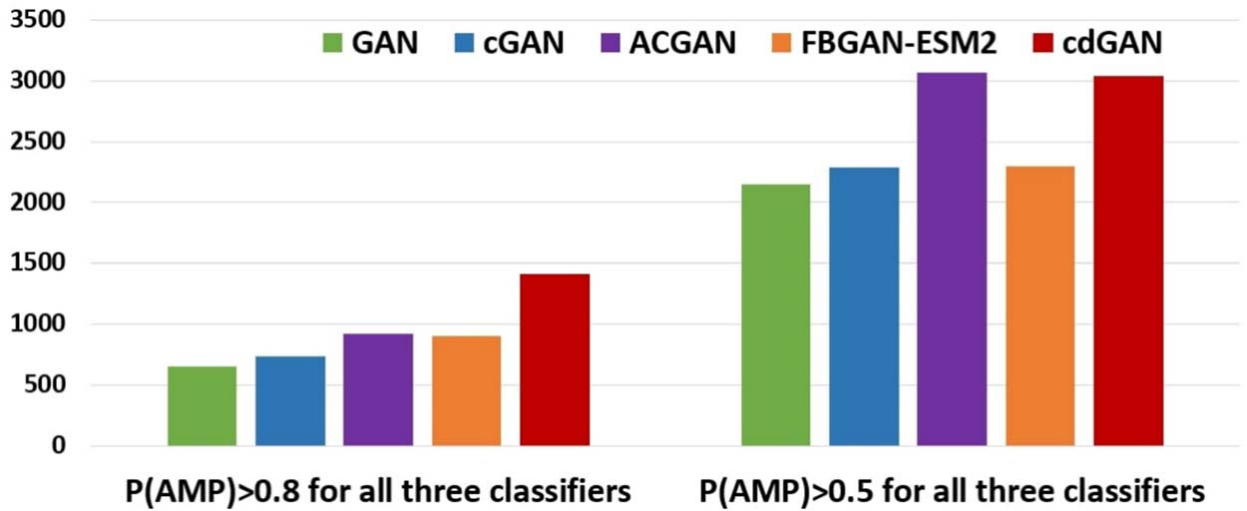
Fraction of generated peptides classified as positive by every classifier of CAMP$_{R4}$ (RF, SVM, and ANN) with probabilities P(AMP)>0.5 (right) and P(AMP)>0.8 (left), respectively.

Figure 4 illustrates the number of sequences predicted as AMPs by all three classifiers, categorized by the thresholds P(AMP) > 0.5 and P(AMP) > 0.8. At the lower threshold, ACGAN and cdGAN emerge as top performers, generating the largest number of predicted AMPs, followed by FBGAN-ESM2, cGAN, and GAN. When considering the stricter threshold, cdGAN retains strong performance, while the rest of the models experience notable declines. Overall, these results demonstrate that cdGAN achieves the best trade-off between diversity, sequence similarity, and classifier-predicted functionality, making it an effective model for AMP design.

### Comparison with state-of-the-art AMP design models

To further assess the potential of the proposed cdGAN model we compare it against state-of-the-art AMP design models, namely, AMPGAN [39], HydrAMP [6], and RLGen [7]. AMPGAN uses bidirectional conditional GANs [40], conditioning on diverse AMP attributes, such as pathogen specificity, mechanisms of action, and minimum inhibitory concentration (MIC), to tailor generated peptides. RLGen is a reinforcement learning-guided generative adversarial network (GAN) framework that leverages a thermodynamic diffusion process to better simulate AMP conformations, while HydrAMP leverages conditional variational auto-encoders [41] to generate peptides based on properties like antimicrobial activity and MIC.

The sequence similarity metric presented in Table 5 reveals that HydrAMP achieves the lowest similarity (21.7%), outperforming even the real data (22.9%) and indicating its superior ability to generate highly diverse sequences. AMPGAN (26.8%) and cdGAN (27.4%) exhibit moderate levels of similarity, whereas RLGen performs the worst (36.9%), indicating reduced diversity in its generated sequences. In terms of the percentage of unique sequences, all models besides HydrAMP achieve no duplicated outputs.

**Table 5:** Percentage of diversity and sequence similarity per model

	In-between Sequence Similarity	Diversity
Real data	22.9%	100%
AMPGAN	26.8%	100%
HydrAMP	**21.7%**	98.9%
RLGen	36.9%	100%
cdGAN (This work)	27.4%	100%

**Figure 5:**
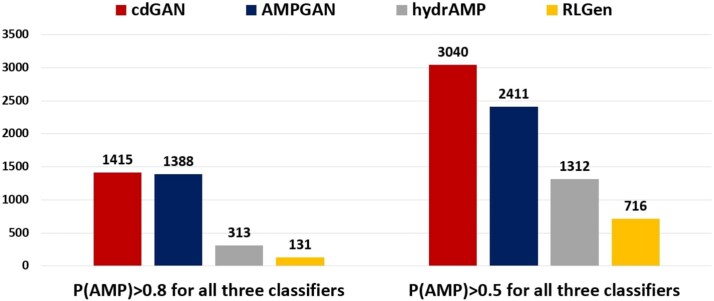
Fraction of generated peptides classified as positive by every classifier of CAMP$_{R4}$ (RF, SVM, and ANN) with probabilities P(AMP)>0.5 (right) and P(AMP)>0.8 (left), respectively.

The CAMP$_{R4}$ predictive results provided in Table 6 show that cdGAN consistently outperforms all other models across all classifiers (RF, SVM, and ANN) highlighting the model’s ability to generate sequences that are both diverse and biologically relevant. Figure 5 demonstrates the number of sequences predicted as AMPs by all three classifiers, categorized by the thresholds P(AMP) > 0.5 and P(AMP) > 0.8. At both thresholds, cdGAN outperforms the other models by generating the highest number of predicted AMPs. However, under the stricter threshold, AMPGAN closely follows in performance.

**Table 6:** Percentage of predicted AMPs with CAMP$_{R4}$ platform equipped with Support Vector Machine (SVM), Random Forest (RF), and Artificial Neural Network (ANN) models for AMP prediction. The best performance per classifier is highlighted in bold, and the standard deviation is reported in parentheses

	CAMP$_{R4}$ predictions			
	RF	SVM	ANN	Average (std)
AMPGAN	64.6%	53.8%	56.6%	58.3% (4.5)
HydrAMP	58.8%	52.6%	32.9%	48.1% (11.0)
RLGen	34.7%	21.1%	21.7%	25.8% (6.2)
cdGAN (This work)	**75.8%**	**68.6%**	**70.5%**	**71.6% (3.0)**

This analysis highlights that cdGAN and AMPGAN are the leading models in generating predicted AMPs with high confidence. cdGAN demonstrates an advantage in generating sequences under broader confidence criteria, while AMPGAN remains competitive at stricter thresholds. HydrAMP and RLGen, in contrast, face challenges in producing sequences that consistently meet high-confidence criteria.

#### Physicochemical properties and amino acid composition

To evaluate the quality and functional relevance of the generated peptides, we analysed four physicochemical properties [42] of AMP sequences with P(AMP) > 0.8 across all classifiers, for each model. Particularly, we evaluate the distribution of charge, isoelectric point (pI), aromaticity, and hydrophobicity ratio. The charge of AMPs plays a crucial role in their interaction with microbial membranes, and the distribution of charge among the generated sequences provides important insights into how well the models replicate this fundamental characteristic. The isoelectric point (pI) distribution, at physiological pH, for the real data has a wide distribution of pI values, with a median around 10, reflecting the prevalence of cationic peptides among AMPs, a key feature for membrane-targeting functionality. Aromaticity is also a crucial feature in AMPs as it can influence their interaction with microbial membranes and overall efficacy. Finally, hydrophobicity is a key factor in shaping the functional properties of AMPs, as it affects their interaction with microbial membranes.

**Figure 6:**
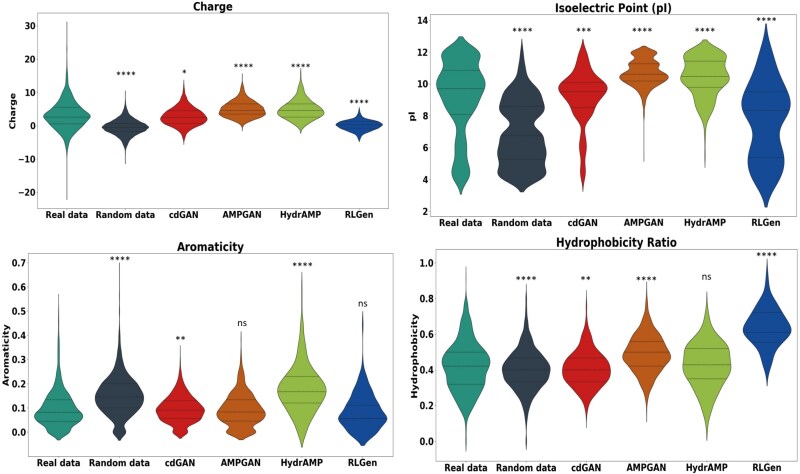
Distribution of physicochemical features such as charge, isoelectric point, aromaticity, and hydrophobicity, of real, random, and generated data per model. Statistical significance was evaluated using the two-sided Mann–Whitney–Wilcoxon test, with p-values denoted as: ns for non-significant values ($p> 0.05$), $^{*} (p \le 0.05)$ indicating a modest level of significance, $^{**}(p \le 0.01)$ indicating stronger significance, $^{***} (p \le 0.001)$ indicating very strong significance, and $^{****} (p \le 0.0001)$ indicating the highest level of significance.

The violin plots in Fig. 6 present the distribution of these four physicochemical properties for AMP sequences with a predicted confidence of $P(AMP)> 0.8$. The properties of sequences generated by the cdGAN model are compared against real data, random data, AMPGAN, HydrAMP, and RLGen. Statistical significance was evaluated using the two-sided Mann–Whitney–Wilcoxon test [43], a non-parametric method suitable for comparing independent samples without assuming normality. No multiple testing corrections were applied, as the number of statistical comparisons was limited.

As presented, the distribution of physicochemical features reveals that cdGAN-generated sequences most closely resemble real AMPs. For charge and pI, cdGAN produces distributions that, while statistically different from real data (p $\le $ 0.05 and p $\le $ 0.001, respectively), are more balanced compared to other models.

In terms of aromaticity, cdGAN-generated peptides show a significant but moderate deviation from real data (p $\le $ 0.01), while AMPGAN’s and RLGen’s results are not significantly different (ns), suggesting better alignment in this specific feature.

For hydrophobicity, cdGAN again demonstrates moderate deviations (p $\le $ 0.01), whereas HydrAMP shows no significant difference from real data, and models like AMPGAN and RLGen exhibit more pronounced differences (p $\le $ 0.0001), indicating less biologically relevant outputs.

Overall, while AMPGAN and HydrAMP demonstrate partial success in replicating specific physicochemical properties, cdGAN offers more balanced results across all features.

## Multi-task cdGAN performance

To demonstrate the effectiveness of the proposed cdGAN model in multi-task design, we present a case study focused on the design of AMPs with specific $\alpha $-helical structure. The ability to generate AMPs with desired structural properties is crucial for enhancing their efficacy and stability, contributing to the development of new therapeutic agents.

Table 7 provides the percentage of predicted AMPs with CAMP$_{R4}$ platform, as well as the percentage of the helical structures predicted with the proposed classifier. The results show that both the single-task and multi-task cdGAN models yield relatively high AMP scores across all classifiers from the CAMP$_{R4}$ platform. In particular, the multi-task cdGAN model consistently produces AMP sequences with an average score of 71.1%, with a standard deviation of 1.7%.

**Table 7:** Percentage of predicted AMPs and $\alpha $-helical structure. The standard deviation is reported in parentheses

	CAMP$_{R4}$ predictions				Helical structure prediction
	RF	SVM	ANN	Average	
					
cdGAN (single-task)	75.8%	68.6%	70.5%	71.6% (3.0)	21.1%
cdGAN (multi-task)	73.4%	69.3%	70.6%	71.1% (1.7)	27.0%

Moreover, the multi-task cdGAN outperforms the single-task cdGAN, achieving 27.0% helical content compared to 21.1% for the single-task version. This suggests that multi-task learning, by optimizing for both AMP activity and helical structure, leads to better generation of helical peptides. These results support the notion that multi-task models can better capture complex relationships between multiple properties.

To evaluate the dual-objective capability of each generative model considering a more realistic peptide design scenario, we first selected sequences classified as AMPs with a probability greater than 95% across all three classifiers of $\text{CAMP}_{R4}$. These sequences were subsequently evaluated for $\alpha $-helical content using our proprietary helix prediction classifier, retaining only those with a helix probability exceeding 95% as well. This stringent threshold ensures that only the most reliable and biologically meaningful candidates are selected for analysis, aligning with the ultimate goal of designing effective therapeutic agents.

The reason we rely on our helix prediction classifier, rather than other methods, is its higher accuracy compared to other related works that classify the structure of AMP sequences [32]. Other existing generic classifiers for secondary structures lack the precision required for AMP validation and achieve lower performance, in general, [16, 29–31]. By employing our classifier, we ensure that the validation of generated sequences aligns with the specialized requirements of AMP research, offering a robust and accurate assessment of their $\alpha $-helical content. The liability of our classifier is also validated with AlphaFold 3 [44] server with the results depicted in Fig. 8.

Table 8 offers insights into each model’s capability to generate sequences that are both AMP and possess a helical structure with high confidence (P(AMP>0.95) and P(helix>0.95)). As shown, AMPGAN demonstrates the highest count of sequences meeting both objectives. This notable performance likely stems from the fact that AMPGAN incorporates more biological knowledge into its design, enabling it to generate sequences that are not only likely to be AMPs but also possess the necessary structural characteristics, such as $\alpha $-helical content. In contrast, other models, including cGAN, ACGAN, FBGAN-ESM2, and HydrAMP, show a remarkably lower number of dual-objective sequences, with HydrAMP failing to generate any qualifying sequences. This underperformance could be attributed to differences in training objectives or architectural limitations that prevent these models from effectively capturing both AMP activity and $\alpha $-helical structural features. RLGen performs slightly better, as its training on thermodynamic principles inherently considers the interdependence between amino acids, which indirectly incorporates structural information, but still falls short of expectations. The relatively limited performance of these models highlights the inherent challenges in designing generative frameworks capable of optimizing multiple complex properties simultaneously.

**Table 8:** Number of sequences generated by each model that meet dual objectives of high AMP probability (P(AMP)> 0.95) and high $\alpha $-helical content probability (P(helix)> 0.95)

	Number of sequences with P(AMP)>0.95 and P(helix)>0.95
GAN	1
cGAN	1
ACGAN	1
FBGAN-ESM2	2
AMPGAN	**57**
HydrAMP	0
RLGen	6
cdGAN (single-task)	10
cdGAN (multi-task)	21

Considering the proposed cdGAN model, in its multi-task configuration, generates 21 sequences meeting the dual objectives, doubling the count achieved by its single-task counterpart that produces 10 sequences.

**Figure 7.**
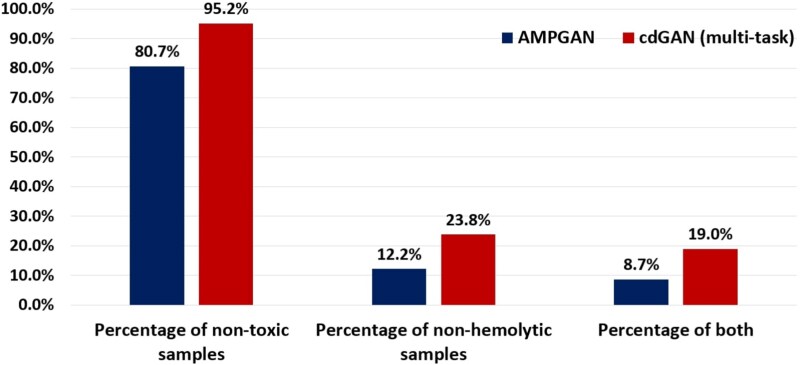
Performance of AMPGAN and cdGAN (multi-task) models based on the percentage of generated AMPs with $P(AMP)>0.95$ and $P(helix)>0.95$, which are non-toxic, non-hemolytic, and satisfy both the previous conditions.

The toxicity of these top-scored sequences is also evaluated with ToxinPred 3.0 [10] to assess their potential for therapeutic applications. Ideally, AMPs that are effective at fighting infections should also exhibit low toxicity to human cells, making them more suitable for development into safe and effective treatments [45]. The results, presented in Fig. 7, demonstrate that 95.2% of the sequences generated by the cdGAN model are non-toxic, compared to 80.7% for AMPGAN, indicating a significant improvement in safety. Similarly, the hemolytic potential of these sequences is assessed using the HemoPI tool [11]. Hemolytic AMPs are less desirable for therapeutic use as they can damage red blood cells, posing a risk of hemolysis. The results show that 23.8% of the cdGAN-generated sequences are non-hemolytic, nearly twice as many as the 12.2% achieved by AMPGAN. Furthermore, 19.0% of the cdGAN sequences meet both criteria of being non-toxic and non-hemolytic, a marked improvement over the 8.7% achieved by AMPGAN. These results highlight the ability of cdGAN’s multi-task learning framework in generating sequences with lower predicted toxicity and hemolysis, making them better suited for therapeutic applications.

**Figure 8:**
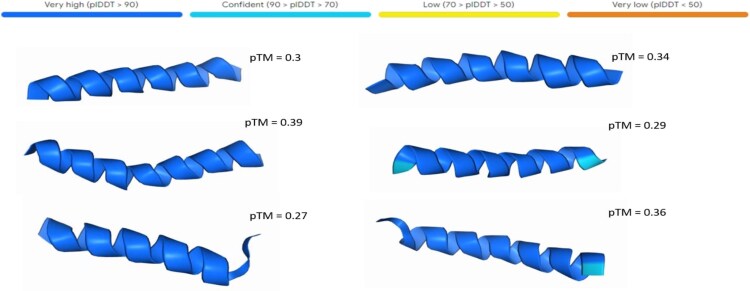
AlphaFold3 structural prediction of peptides generated by the cdGAN model, with probabilities P(AMP)>0.95 and P(helix)>0.95. The predicted Template Modeling (pTM) and per-residue local distance difference test (pLDDT) scores are also provided.

Figure 8 presents AlphaFold3 [44] structural predictions for cdGAN-generated AMPs with high predicted antimicrobial activity ($P(\mathrm{AMP})> 0.95$) and strong $\alpha $-helical likelihood ($P(\mathrm{helix})> 0.95$). The per-residue local distance difference test (pLDDT) scores are exceptionally high across all cases (ranging from 80 to 95), demonstrating a high degree of confidence in the overall structural quality. The predicted Template Modeling (pTM) scores for the peptides range from 0.27 to 0.39. Although these are below the 0.5 threshold typically associated with high-confidence global folds, this is expected in de novo design, where the sequences are novel and not derived from known structural templates [46]. These moderate scores suggest the peptides may adopt coherent, protein-like structures despite lacking homology to known proteins. However, the low pTM scores also reflect limited confidence in the predicted global folds, and these structural predictions should therefore be interpreted with caution.

## Discussion

The findings of this study highlight the multifaceted nature of AMP generation, emphasizing the balance between productivity, functional relevance, and physicochemical fidelity. Among the evaluated models, cdGAN emerges as a robust performer, excelling across multiple dimensions and achieving a balance that other models struggle to replicate.

The analysis of physicochemical properties further emphasizes cdGAN’s strengths. Its ability to replicate the charge and pI distributions of real AMPs, while maintaining hydrophobicity levels consistent with natural variability, reflects a nuanced understanding of AMP characteristics. This is statistically supported by the results of the Mann–Whitney–Wilcoxon test, where cdGAN-generated peptides demonstrated significant similarity to real data in terms of charge ($p \le 0.05$) and hydrophobicity ($p \le 0.01$). In contrast, AMPGAN and HydrAMP generate peptides with elevated charge profiles, suggesting a potential bias toward membrane-targeting functionality, which, while useful for specific applications, may limit the overall diversity. Similarly, RLGen showed statistically significant deviations ($p \le 0.0001$) from real AMPs, exhibiting overly broad, misaligned property ranges. These findings underscore the importance of balancing functional optimization with fidelity to real AMP profiles, a challenge that cdGAN addresses effectively.

The multi-task learning framework employed by cdGAN demonstrates a clear advantage in optimizing for multiple objectives simultaneously. In its multi-task configuration, cdGAN produces a higher proportion of sequences exhibiting both AMP activity and $\alpha $-helical structure, outperforming its single-task counterpart doubling the count of sequences meeting these dual objectives.

The toxicity and hemolytic analysis further underscores the therapeutic potential of multi-task cdGAN-generated peptides. Among high-confidence AMP sequences ($P(AMP)> 0.8$), 95.2% of the sequences generated by cdGAN are classified as non-toxic, compared to 80.7% for the competitive AMPGAN model. This represents a significant improvement in toxicity reduction, highlighting cdGAN’s superior ability to generate therapeutically viable peptides. Similarly, in the hemolysis analysis conducted with the HemoPI tool, nearly double of the cdGAN sequences were classified as non-hemolytic, compared to AMPGAN’s and more the double satisfied both criteria (non-toxic and non-hemolytic). These findings showcase cdGAN’s ability to balance structural functionality, therapeutic safety, and biological relevance.

The structural predictions utilizing AlphaFold3 further emphasize the capabilities of the multi-task cdGAN model in generating de novo AMPs. Specifically, the exceptionally high per-residue local distance difference test (pLDDT) scores ($>90$) indicate a high degree of confidence in the structural predictions. Additionally, the predicted Template Modeling (pTM) scores, ranging from 0.27 to 0.39, are consistent with the expectations for de novo design, where the sequences are not based on existing structural templates. These results validate the model’s ability to generate peptides with $\alpha $-helical structure, which is essential for membrane disruption and antimicrobial efficacy.

In summary, while models like AMPGAN, HydrAMP, and RLGen demonstrate strengths in specific aspects, they fail to replicate the comprehensive balance achieved by cdGAN. The ability of cdGAN to simultaneously optimize for antimicrobial activity, structural relevance, physicochemical fidelity, toxicity, and hemolytic potential underscores its versatility and effectiveness as a design tool for therapeutic peptide discovery.

**Key Points**

- A classifier-driven GAN framework (cdGAN), which integrates classifier predictions directly into the loss function, allowing for dynamic and adaptive AMP generation.
- Extension of cdGAN to a multi-task setting, incorporating both antimicrobial activity and structural optimization using a novel ESM2-based multi-task classifier.
- Enhance peptide safety by performing post-generation toxicity and hemolysis screening, ensuring that generated peptides are both effective and viable for therapeutic applications.

## Supplementary Material

supplementary_revised_bbaf500

## Data Availability

The datasets and code used in this study are publicly available at https://github.com/aretiz/amp_de_novo_design_cdGAN.
