# Supplementary material for: Classifier-driven generative adversarial networks for enhanced antimicrobial peptide design
Source: Brief Bioinform. 2025 Oct 25;26(5):bbaf500. doi: 10.1093/bib/bbaf500 (PMC12553139; doi:10.1093/bib/bbaf500)
Supplement: supplementary_revised_bbaf500 [file supplementary_revised_bbaf500.pdf]

Supplementary Material for: *"Classifier-driven Generative Adversarial Networks for Enhanced Antimicrobial Peptide Design"*

Michaela Areti Zervou, Effrosyni Doutsi, Yannis Pantazis, Panagiotis Tsakalides

In this study, each framework is developed using PyTorch, an open-source machine learning framework in Python, on a desktop computer equipped with NVIDIA's GPU model GeForce RTX 3070.

1 Classifier Setup and Performance

This study introduces a multi-task classifier designed to assess two key properties of protein sequences: (i) antimicrobial potency, and (ii) the likelihood of an α-helical fold. Transfer learning is performed using ESM2 , a protein language model trained on an extensive dataset of protein sequences, which enables it to capture intricate sequence patterns and features. The ESM2-t12 variant, a 12-layer Transformer-based model with 35 million parameters, was selected for its optimal balance between embedding size and parameter count, ensuring computational efficiency without compromising on sequence embedding quality. In contrast, the ESM2-t33 variant, with 650 million parameters, requires significantly more computational resources.

The classifier is implemented as a two-layer multilayer perceptron (MLP), applied to the embeddings obtained from the frozen ESM2 encoder. The first layer uses ReLU activation followed by dropout to mitigate overfitting, while the second layer outputs the final classification logits. The model is trained using the Adam optimizer with fixed hyperparameters $\beta_1 = 0.9$ and $\beta_2 = 0.999$, and the cross-entropy loss function. Training is performed over 30 epochs, and the model with the lowest validation loss is retained for final evaluation.

To optimize classifier performance, we performed a grid search over key hyperparameters including the number of hidden units, learning rate, dropout rate, and batch size. The data were stratified and split into three disjoint sets: 60% for training, 20% for validation, and 20% for testing. The classification procedure was repeated 20 times using different random seeds for data splitting, and we report the average performance along with standard deviation across these runs.

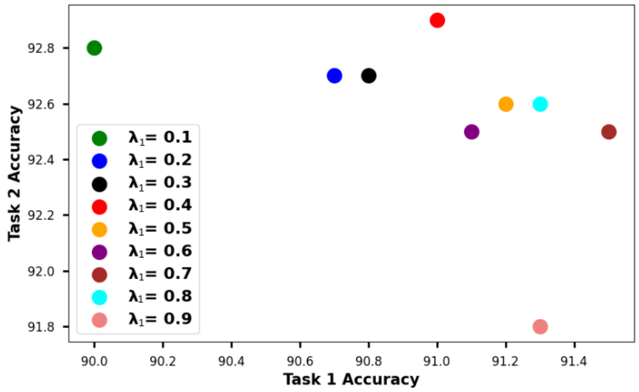

Figure 1: Average classification accuracy for various values of the regularization parameter λ_1 ($\lambda_2 = 1 - \lambda_1$) in detecting AMP (Task 1) and α-helical folding properties (Task 2) using the proposed MLP-ESM2 classifier.

Table 1: Effect of λ_1 values ($\lambda_2 = 1 - \lambda_1$) in multi-task classification of AMP (Task 1) and α-helical folding prediction (Task 2) using the proposed MLP-ESM2 classifier.

	λ_1=0.1		λ_1=0.2		λ_1=0.3	
	Task 1	Task 2	Task 1	Task 2	Task 1	Task 2
**Accuracy**	90.0 (1.0)	92.8 (0.8)	90.7 (0.7)	92.7 (0.4)	90.8 (0.8)	92.7 (0.4)
**AUC**	96.1 (0.6)	98.0 (0.3)	96.3 (0.6)	98.0 (0.2)	96.6 (0.6)	98.1 (0.2)
**F1-score**	90.0 (0.8)	93.4 (0.8)	90.6 (0.7)	93.3 (0.4)	90.9 (0.8)	93.3 (0.4)

	λ_1=0.4		λ_1=0.5		λ_1=0.6	
	Task 1	Task 2	Task 1	Task 2	Task 1	Task 2
**Accuracy**	91.0 (0.9)	92.9 (0.5)	91.2 (0.7)	92.6 (0.6)	91.1 (0.7)	92.5 (0.4)
**AUC**	96.8 (0.6)	98.1 (0.2)	96.8 (0.6)	98.0 (0.2)	96.8 (0.6)	98.0 (0.2)
**F1-score**	91.1 (0.8)	93.5 (0.4)	91.2 (0.7)	93.2 (0.6)	91.1 (0.7)	93.2 (0.4)

	λ_1=0.7		λ_1=0.8		λ_1=0.9	
	Task 1	Task 2	Task 1	Task 2	Task 1	Task 2
**Accuracy**	91.5 (0.9)	92.5 (0.7)	91.3 (0.7)	92.6 (0.8)	91.3 (0.5)	91.8 (0.7)
**AUC**	96.9 (0.6)	98.0 (0.2)	96.9 (0.5)	97.9 (0.2)	96.9 (0.5)	97.7 (0.3)
**F1-score**	91.6 (0.9)	93.1 (0.6)	91.4 (0.8)	93.3 (0.8)	91.3 (0.5)	92.6 (0.7)

The final classifier uses an input embedding size of 480 and consists of two fully connected layers with 128 hidden units. A learning rate of 0.001, a dropout rate of 0.2, and a batch size of 64 were selected based on validation performance. The total number of parameters is approximately 61.8K, making the classifier efficient and computationally lightweight.

The analysis metrics, including accuracy, AUC, and F1-score, for each λ_1 and λ_2 values (where $\lambda_2 = 1 - \lambda_1$) are presented in Table 1. As shown, the

performance for both Task 1 and Task 2 remains closely aligned across all λ_1 values, with only minor differences observed, suggesting that the model is relatively insensitive to variations in the task weighting parameter. This close performance across all λ_1 values implies a degree of interdependence between Task 1 (AMP classification) and Task 2 (α-helical folding prediction), likely due to shared feature utility derived from the ESM2 embeddings.

Figure 1 illustrates the classification accuracy of the proposed MLP-ESM2 classifier across a range of λ_1 values for the multi-task classification of AMP detection (Task 1) and α-helical folding prediction (Task 2). A Pareto front is revealed, where specific λ_1 values, such as λ_1=0.4 (red), λ_1=0.8 (cyan), and λ_1=0.7 (brown), achieve high accuracy in both tasks. We select λ_1=0.4 as the optimal that balances both tasks. Nevertheless, as already mentioned, the choice of λ_1 has minimal impact on the overall classification performance, allowing for robust classification regardless of the shifts in task prioritization.

2 cdGAN, ACGAN and cGAN Architecture and Training Procedure

To ensure a fair and consistent comparison across all generative models, we first optimized a baseline GAN architecture by extensively tuning key hyperparameters such as hidden dimensionality, learning rate, batch size, and training dynamics. This process yielded a stable and expressive architecture, which we then adopted as a common backbone for all model variants: cdGAN, cGAN, and ACGAN. Our study is explicitly designed to isolate and evaluate the impact of different conditioning strategies, such as continuous conditioning, discrete label conditioning, and auxiliary classification, by holding the architecture constant. This approach eliminates confounding factors and allows us to directly attribute differences in performance to the integration of the classifier and the specific conditioning mechanisms, which is the central focus of our investigation. Specifically, each model employs fully connected generator and discriminator networks with a hidden dimensionality of 512, optimized using the Adam optimizer with a learning rate of 0.0001 and a batch size of 64. Training proceeds for 150 epochs, where the discriminator is updated ten times per generator step. Sequences are constrained to a maximum of 156 nucleotides, and the generator's output is decoded using a Gumbel-Softmax operation with a temperature of 0.75 followed by argmax sampling. The Wasserstein loss with gradient penalty (coefficient 10) is employed, replacing the traditional SoftMax, allowing for stochastic sampling during sequence generation. At each epoch, we sample sequences from the generator, using the argmax of the probability distribution to output a single nucleotide at each position. After generating the sequences, we employ our pretrained classifier to assess their quality and biological relevance.

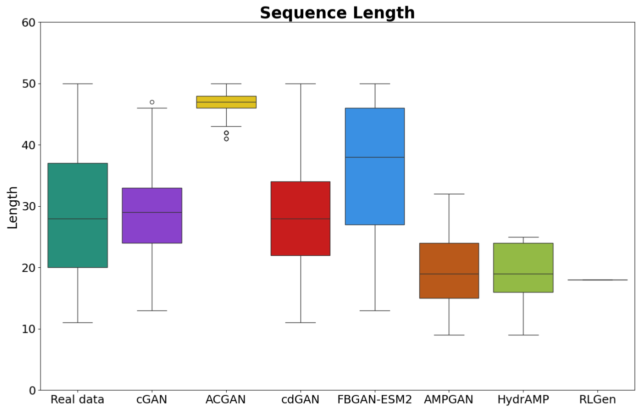

Figure 2: Sequence length distribution of real and generated samples per model.

3 Sequence length of generated peptides

Figure 2 illustrates the distribution of sequence lengths for real and generated data across various models, highlighting their ability to replicate the natural variability observed in real sequences. Real data exhibit a length distribution spanning approximately 20 to 40 residues, with a median length of about 30 residues. The distribution also includes outliers extending to 50 residues, reflecting the inherent diversity of the dataset.

Among the generative models, FBGAN-ESM2 demonstrates the highest variability, with most generated sequences clustering around 40 amino acids. While this suggests an attempt to capture variability, the distribution is less aligned with the real data compared to other models. cGAN and cdGAN, in contrast, produce sequences with narrower distributions, with median lengths closely resembling real data. This indicates that these models are able to preserve some of the natural characteristics of sequence length but may compromise diversity in doing so. In comparison, ACGAN, AMPGAN, and HydrAMP show even more constrained distributions. Notably, ACGAN generates sequences tightly clustered around a median length of 50 residues, with minimal variation, suggesting overfitting or excessive regularization during training. Similarly, AMPGAN and HydrAMP generate shorter sequences with limited variability, focusing on specific length ranges but failing to capture the full diversity of real data. RLGen, on the other hand, generates sequences with a fixed length of 20 amino acids. While this could be beneficial for applications requiring uniform lengths, it significantly restricts the model's flexibility and generalizability.

4 Normalized edit distance

Figure 3 presents the normalized edit distance analysis for state-of-the-art AMP design models. The distribution of data generated by cdGAN closely aligns

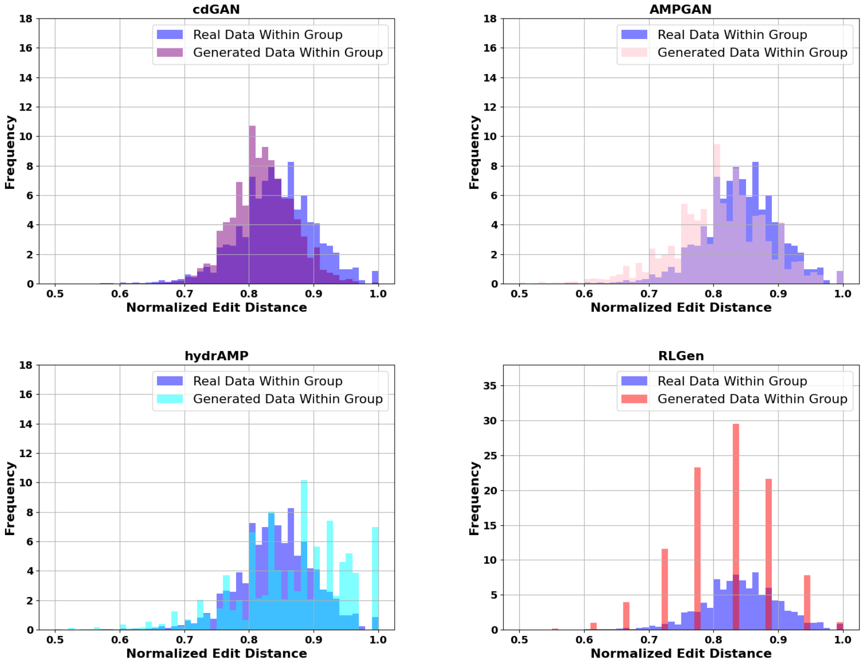

Figure 3: Normalized within group edit distance distribution for real and generated data, highlighting the fidelity and diversity of different models.

with that of real data, indicating high fidelity. AMPGAN, while demonstrating strong diversity, exhibits a broader distribution that is slightly shifted from the real data, suggesting lower fidelity compared to cdGAN. HydrAMP also produces highly diverse peptides but with reduced fidelity. Lastly, RLGen shows the greatest divergence, generating distributions that are significantly broader and less centered, highlighting its difficulty in accurately capturing the variability of real peptides.

5 Amino acid composition and physicochemical properties

The amino acid composition and physiochemical properties of the top-performing AMPs (P(AMP) $>$ 0.8 across all classifiers) for each model.

The analysis of amino acid composition across each model is also provided in Figure 4. When compared to real protein sequences, each model exhibits unique amino acid frequency distributions, suggesting differential capture of sequence features. Models like AMPGAN and HydrAMP demonstrate elevated frequencies of amino acids K and R, potentially indicating a focus on sequences

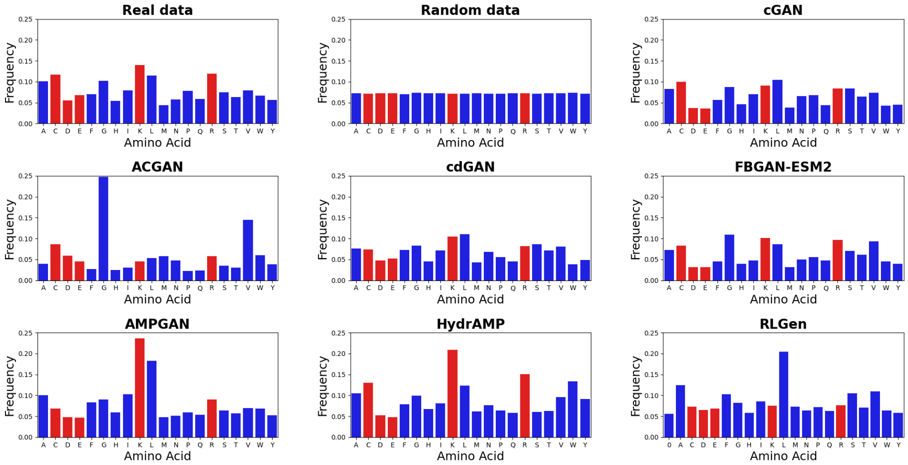

Figure 4: Amino acid composition of real, random and generated data per model. Red bars indicate amino acids contributing to charge, hydrophobicity, amphiphilicity, and secondary structure.

Table 2: Kullback–Leibler Divergence between the amino acid compositions of real and generated protein sequences per model.

	Kullback–Leibler Divergence
cGAN	0.056
ACGAN	0.445
FBGAN-ESM2	0.058
AMPGAN	0.196
HydrAMP	0.166
RLGen	0.231
cdGAN (This work)	**0.027**

with higher positive charge or roles in electrostatic interactions.

The Kullback–Leibler Divergence (KLD) presented in Table 2, is used to measure the statistical divergence between the amino acid compositions of real and generated protein sequences. Specifically, among the models evaluated, cdGAN (0.027), cGAN (0.056), and FBGAN-ESM2 (0.058) demonstrate the best performance, generating sequences that closely align with the real data in terms of amino acid distribution. In contrast, models such as ACGAN (0.44) exhibit higher KLD scores, indicating that their generated sequences diverge more significantly from the real sequences' amino acid composition. Other models, including AMPGAN (0.19), HydrAMP (0.16), and RLGen (0.23), show moderate divergence, suggesting that while their generated sequences share some similarities with real sequences, they still exhibit notable differences in amino acid distribution.

6 Diversity and sequence similarity of multi-task cdGAN

Table 3: Percentage of diversity and sequence similarity per model.

	In-between Sequence Similarity	Diversity
Real data	22.9%	100%
cdGAN (single-task)	27.4%	100%
cdGAN (multi-task)	28.5%	100%

Table 3 compares the sequence similarity and diversity of AMPs generated by cdGAN in single-task and multi-task configurations against real data. Both models of cdGAN achieved 100% diversity indicating their ability to generate a wide variety of unique sequences. The sequence similarity is slightly increased for cdGAN multi-task (28.5%) compared to the single-task counterpart (27.4%).
